# When severe violence becomes a script: a vulnerability-script integration model of media exposure and the form of psychosis-associated maternal filicide

**DOI:** 10.3389/fpsyt.2026.1993972

**Published:** 2026-10-27

**Authors:** Changiz Mohiyeddini

**Affiliations:** 1 Michael Garron Hospital, Toronto East Health Network, Toronto, ON, Canada; 2 Department of Family and Community Medicine, Temerty Faculty of Medicine, University of Toronto, Toronto, ON, Canada

**Keywords:** aggressive scripts, causal inference, copycat crime, delusions, forensic psychiatry, maternal filicide, media contagion, elective exposure

## Abstract

When a publicized act of severe violence is followed by one that resembles it, the resemblance is often read as contagion. This paper argues that resemblance is weak evidence of influence and asks a narrower question: under what conditions can exposure to a publicized crime narrative shape the form of subsequent violence in a person whose antecedent state has already made such violence conceivable? The Vulnerability-Script Integration Model (VSIM) is developed for psychosis-associated maternal filicide. It is an organizing framework with an attached evidentiary protocol and three new propositions: antecedent vulnerability selects exposure as well as moderating its effect; exposure primarily shapes the form rather than the origin of violence; and media influence is inferred only from a dated post-exposure change in narrative-specific behavioral information (acquired or activated) that the documented antecedent state does not parsimoniously explain. The model specifies five functional stages, two moderators, a clinically defined and non-circular vulnerability construct, discriminant coding rules, a causal diagram with explicit estimands, and six predictions with stated classification, test, disconfirming, and inconclusive evidence. A closed, adjudicated case in which a court-tested claim of media scripting proved false illustrates the evidentiary rules. Clinical and reporting implications are framed as research questions.

## 1 Introduction

In 2002, a Texas jury rejected the insanity defense of Andrea Yates, who had drowned her five children in June 2001 after years of documented psychotic illness. The State’s sole mental health expert testified that an episode of the television drama Law and Order, on which he consulted, had depicted a woman who drowned her children and was acquitted by reason of insanity, and that it had aired shortly before the killings; the prosecution used the testimony in cross-examination and closing argument to suggest that Yates had patterned her act on the program. No such episode existed. The Texas Court of Appeals reversed the conviction in 2005 because the false testimony was material to the only expert opinion supporting the State’s case, and a second jury found Yates not guilty by reason of insanity in 2006 (1, 2). The case is examined in Section 8. It is cited here because it displays, in a closed record, the two things this paper is about: the intuitive force of the claim that a publicized narrative shaped a violent act, and the absence of any evidentiary standard by which such a claim is ordinarily tested.

The intuitive claim is usually put as “why do serious crimes create a model that others follow?” That phrasing assumes its answer. It presupposes a causal channel from the first act to the second and invites the vocabulary of copycat killing, in which a publicized act is treated as sufficient to produce its repetition. The term copycat is used here only when reporting others’ usage, because it names an outcome rather than a mechanism. The scientific question is narrower: under what conditions can exposure to a publicized crime narrative supply a behavioral script that shapes the form of subsequent violence in a person whose antecedent state has already made such violence conceivable?

The reframing follows Surette (3, 4). Media crime models rarely function as triggers that generate motivation where none existed; they more often function as rudders, supplying technique, form, target, and timing to people whose motivation arose elsewhere. Surette (4) summarizes the offender literature in a phrase that organizes this paper: offenders take instructions from criminogenic media more than they take motivations. If so, the appropriate model is one in which antecedent vulnerability supplies motivational pressure and a salient, self-relevant narrative supplies form.

What this paper offers should be stated plainly. The Vulnerability-Script Integration Model (VSIM) is primarily an organizing framework and an evidentiary protocol, not a new explanatory theory of violence. Its stages adapt established constructs from social cognitive theory, aggressive script theory, selective exposure research, and the cognitive model of delusions. What is new is three propositions and the test they jointly imply. First, antecedent vulnerability selects exposure as well as moderating its effect, so that exposure cannot be treated as an independent variable. Second, exposure primarily shapes the form of violence rather than originating its motivation. Third, because of the first two, resemblance between acts is weak evidence of influence; the inference requires a dated post-exposure change in narrative-specific behavioral information that the documented antecedent state does not parsimoniously explain. A combination of script theory, selective exposure theory, and delusion models predicts that concordance will accompany exposure in vulnerable people. VSIM predicts that this concordance is uninformative, and that only a post-exposure change in narrative-specific material discriminates influence from selection. That is the observation that separates the model from the simpler combination it is built from (Section 6.5).

Sections 2 through 5 review the evidence base and state how it was assembled and what each part can bear. Section 6 specifies the model. Section 7 states estimands, predictions, measurement rules, and discriminant coding. Section 8 applies the rules to the Yates record. Sections 9 through 11 present implications, limitations, and conclusions.

## 2 The evidence base and what it can bear

### 2.1 How the literature was assembled

This is a hypothesis paper and no systematic review was conducted. The literature was assembled in four bodies, each searched in PubMed and PsycINFO to September 2026 with the terms indicated, supplemented by backward citation from the principal reviews: suicide contagion and media effects (Werther, Papageno, media reporting, suicide clusters); mass-killing and copycat crime (contagion, imitation, copycat, mass shooting, media coverage); delusions and violence (acting on delusions, persecutory delusions, schizophrenia and violence, cultural content of delusions); and maternal filicide and postpartum psychosis (filicide, infanticide, neonaticide, postpartum psychosis, insanity). Meta-analyses and national case series were preferred where they exist. The bodies differ in outcome, exposure, population, and level of analysis, and Section 2.6 states which claims each can support.

### 2.2 Suicide: the best-established imitation effect

Phillips (5) reported that front-page coverage of suicides was followed by increases in suicide rates. Gould et al. (6) showed that prominent newspaper coverage, particularly coverage describing method, was associated with initiation of adolescent suicide clusters. Niederkrotenthaler et al. (7) showed the effect is content-dependent: reports describing method are associated with increases, reports portraying coping with decreases. A meta-analysis found that reports of celebrity suicide were followed by a pooled increase of approximately 13% in the subsequent one to two months, with a further increase in suicides by the reported method (8). The effect is modest in population terms, strongest where the observer resembles the person depicted, specific to method, and short-lived.

### 2.3 Mass killings: different estimands

Towers et al. (9) reported temporal clustering of mass killings consistent with contagion. Lankford and Tomek (10) found no clustering beyond chance with a different definition and model. Fox et al. (11) found that shootings increase coverage, but coverage has little detectable short-term effect on shootings. Dubow et al. (12) characterize the population evidence as mixed while noting that publicized shootings evidently serve as models for some individuals. These studies address different estimands and are not directly contradictory: population time-series test whether event rates rise; case-level analyses test whether particular offenders were influenced by particular prior offenders.

### 2.4 Copycat crime

Helfgott (13) found the copycat literature dominated by anecdote. Surette (3) proposed criteria for identifying copycat events (time order, theme consistency, scene specificity, repetitive viewing, offender statements, second-party statements) and reported that a minority of incarcerated offenders describe attempting a crime they had seen depicted, usually in the domain of technique. Surette (4) concludes that copycat crime is more common in property and instrumental offending, where media supply usable instructions, and rarer in expressive violence, where motivation is limiting. Two implications follow: the present model is confined to expressive violence, and Surette’s similarity-based criteria cannot be used both to classify a case as media-influenced and to measure the resemblance that classification is meant to explain (Section 7.3).

### 2.5 Filicide

No study known to the author has tested whether publicized filicide cases are followed by changes in the incidence or form of filicide. Friedman et al. (14) estimated roughly 500 filicides per year in the United States, committed by mothers and fathers at similar rates (15). The low base rate makes population-level tests difficult and places a premium on international registries, pooled data, and case-level evidence.

### 2.6 What each body of evidence supports

Four grades of support are distinguished and used throughout. Direct evidence for a VSIM pathway in filicide: none exists. Evidence for a component mechanism in the relevant population: the delusions-and-violence literature (Section 4) supports the vulnerability construct; the filicide literature supports the existence and clinical characterization of the psychotic pathway; forensic case material, such as the Yates record, supports the incorporation of media content into delusional systems. Analogical evidence from another domain: the suicide and mass-killing literatures support the existence of media imitation effects, their content-dependence, their identification-dependence, and their temporal profile, in domains that differ from filicide in outcome, exposure, and population; their weight here is analogical, and Predictions 5 and 6 are labelled accordingly. Model-generated speculation: the selection, uptake, and appraisal stages as applied to filicide, the discriminant coding rules, and the clinical and reporting considerations are generated by the model and have no evidentiary support beyond coherence with the above.

## 3 From social learning to aggressive scripts

Observational learning (16–18) requires attention, retention, reproduction, and motivation shaped by observed consequences. It explains how a behavior can be acquired from a media model but not why, of the millions who attend to the same coverage, almost none reproduce it. Aggressive script theory (19, 20) locates the limiting factor: scripts are stored representations, acquired through observation and rehearsal, that specify what happens in a situation, which responses are available, and what follows; they are retrieved when cues match encoding; retrieval depends on accessibility, which increases with recency, frequency, vividness, and arousal; and a retrieved script is appraised against normative beliefs and current goals before it guides behavior. The General Aggression Model (21) treats knowledge structures as the primary channel of media influence. Gilbert and Daffern (22) showed that aggressive scripts and violent fantasy overlap, that rehearsal without enactment is self-reinforcing because it reduces negative affect, and that script elaboration is associated with violence in offender samples.

Four consequences follow. Trial coverage, repeated, vivid, emotionally charged, and extended over days, satisfies every condition that raises accessibility; this mechanism is related to, but distinct from, the availability heuristic (23). A retrieved script is not enacted unless it survives appraisal, which for almost all exposed people a filicide script would not. Survival depends on congruence with the person’s existing cognitive and affective state. And a person holding a rehearsed aggressive script is likely to seek congruent material, because rehearsal regulates affect (22); exposure is therefore partly endogenous to vulnerability. Script theory also distinguishes acquisition (the narrative supplies a representation that did not previously exist) from activation (it makes an existing representation more accessible without adding content). These generate different evidence and are kept apart throughout.

## 4 The psychotic pathway

Fazel et al. (24) and Whiting et al. (25) confirm an association between schizophrenia spectrum disorders and violence, with much of the excess attributable to comorbid substance use and with low absolute risk; most people with these disorders are never violent. Buchanan et al. (26) found that acting on a delusion was associated with high conviction, evidence-seeking, and negative affect in response to the belief. Coid et al. (27) and Ullrich et al. (28) found that persecutory delusions predicted violence through anger. Delusions per se are therefore not sufficient predictors of violence; the evidence is strongest for persecutory delusions with anger or threat-related affect, although altruistic and child-focused delusional beliefs characterize a proportion of filicides (29, 30) and follow a logic of protection, obligation, or necessity that must be distinguished.

The form of a delusion is a feature of the illness; its content is drawn from the person’s world (31). Gold and Gold (32) described delusional systems organized around reality television; Collin et al. (33) and Pappa et al. (34) document the cultural variation of delusional themes. The cognitive model (35, 36) holds that delusions arise from anomalous experience interpreted under threat anticipation and reasoning biases and are maintained by safety behaviors and confirmatory attention. A vivid, repeated, thematically matched narrative is the kind of input such a system incorporates; it does not create the psychosis but supplies material the psychosis organizes. The Yates record illustrates this at the level of forensic evidence: the defense expert testified that before the killings she had perceived personal messages from Satan in two feature films (37). Two cautions follow. The belief that a child is the devil, or that a child must die to be saved, is delusional content concerning the child; there is no category of “filicidal delusion.” And shared category membership is not psychological identification.

## 5 Psychosis-associated maternal filicide

Resnick (29) classified filicide as altruistic, acutely psychotic, unwanted child, accidental, or spouse revenge. Friedman et al. (14) found maternal filicide heterogeneous, with mental illness prominent in some categories and not others. Friedman et al. (30), examining 39 mothers found not guilty by reason of insanity, found most had been psychotic and that altruistic motives predominated. Flynn et al. (38) found in a national case series that a substantial minority of perpetrators had a history of mental disorder, often unrecognized before the offense. Frederique et al. (39) emphasized the psychotic versus non-psychotic distinction. Friedman et al. (15) reiterate that most filicides in the general population are fatal maltreatment, whereas among insanity acquittees altruistic and psychotic motives predominate.

Three terms are kept separate throughout. Maternal filicide is a category of act. Psychosis is a clinical state; postpartum psychosis is a subtype defined by onset after delivery, strongly related to bipolar disorder, with delusions frequently concerning the infant and an elevated risk of infanticide when untreated (40, 41). Legal insanity is a jurisdiction-specific standard, not a diagnosis. Maternal filicide and postpartum psychosis are not coextensive: most maternal filicides are not psychotic, and most psychotic maternal filicides are not postpartum in onset. The model is developed for the intersection, psychosis-associated maternal filicide, defined as filicide by a mother in whom a psychotic disorder with delusional content concerning the child was present before the act. Every other filicide category, and every non-psychotic pathway to severe violence, is outside the developed model (Section 6.1).

This domain is theoretically informative because the vulnerability construct can be clinically specified, because trial coverage of such cases is organized around motherhood and the perpetrator’s mental state and so transmits an account as well as an act, and because the published forensic literature on insanity acquittees provides the kind of record the model requires. It is not an easy domain for testing, because the base rate is low.

## 6 The vulnerability-script integration model

### 6.1 Scope, definitions, and pathway strategy

VSIM is developed for psychosis-associated maternal filicide as defined in Section 5. Depressive and grievance pathways to severe violence are named as possible extensions in Section 9.3; nothing in the developed model is claimed for them. VSIM makes no claim about instrumental or property offending. Within scope, the following terms are used in fixed senses. Severe expressive violence: physical violence against a person that is not primarily instrumental to a material or situational goal, in which the act itself expresses the perpetrator’s affective or ideational state; in the developed model, filicide by a psychotic mother. Script: a stored representation specifying a situation, a behavioral sequence, and expected consequences (19). Narrative-specific material: an element of a script that is identifiable with a particular publicized narrative and uncommon in the reference population of similar acts. Form: the method, sequence, setting, and timing of an act, as distinct from its target and its motivation. Behavioral information: any dated trace of ideation, rehearsal, searching, planning, or communication bearing on form.

### 6.2 Architecture

VSIM comprises five functional stages, two moderators acting on specified links, one feedback path, and one class of proximal modifiers (**Figure 1**; **Table 1**). The vertical arrangement depicts hypothesized functional dependencies, not an established temporal order; identification, uptake, and appraisal may interact more dynamically than the diagram suggests.

**Figure 1:**
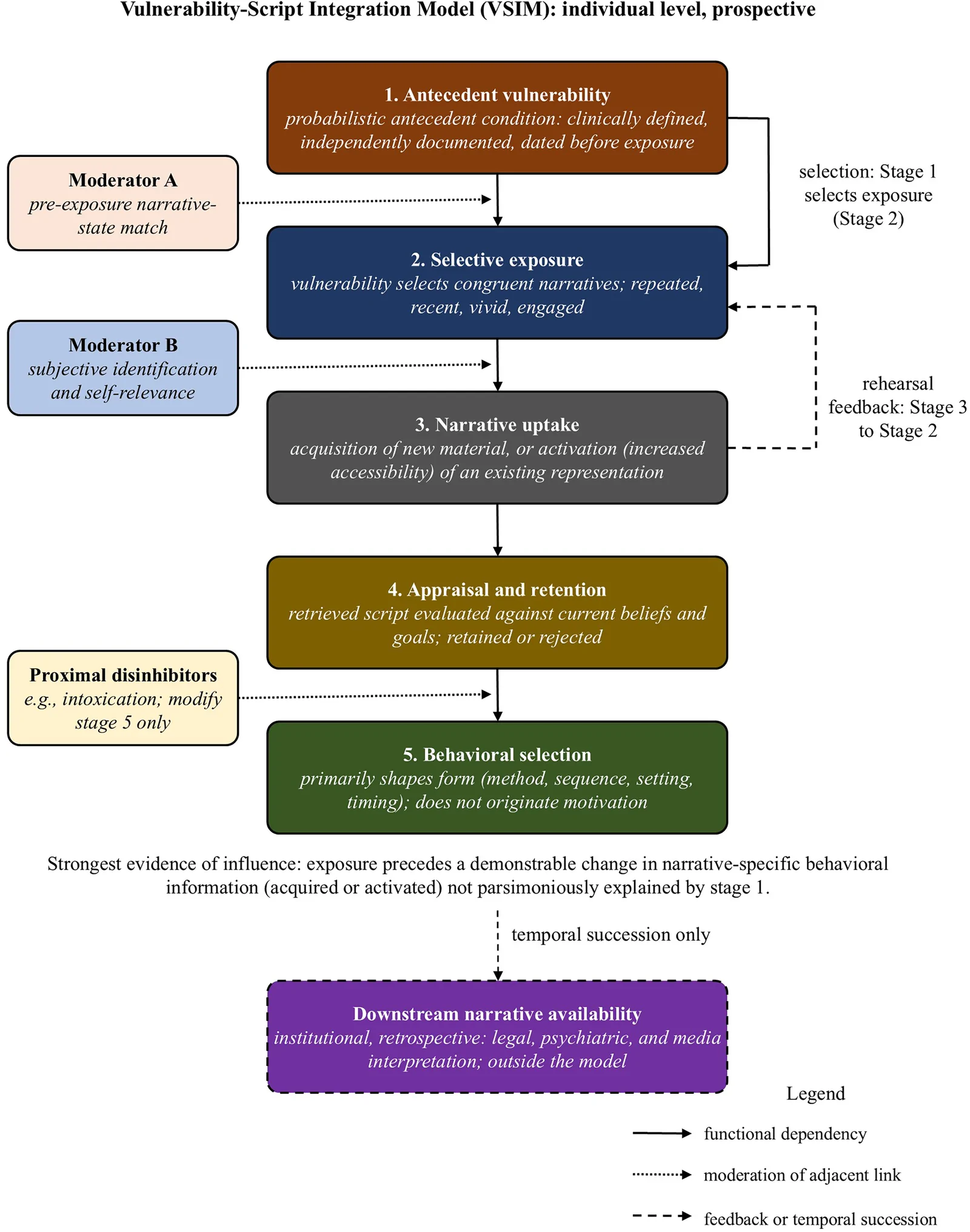
The Vulnerability-Script Integration Model (VSIM). Solid boxes are the five functional stages; solid arrows are hypothesized functional dependencies, not an established temporal order. Dotted arrows show moderation of the adjacent link by pre-exposure narrative-state match (Moderator A), subjective identification (Moderator B), and proximal disinhibitors, which modify Stage 5 only. The unidirectional arrow on the right shows that antecedent vulnerability selects exposure; the model does not propose that exposure changes vulnerability. The dashed arrow from Stage 3 to Stage 2 is rehearsal feedback. Accessibility is a property of Stage 3, changed by activation; Stage 4 is appraisal and retention. The dashed box is downstream narrative availability, an institutional and retrospective process outside the model. The inference criterion of Section 6.3 is stated beneath Stage 5.

**Table 1:** Elements of the vulnerability-script integration model (VSIM): role, proposition, observable indicators, and disconfirming evidence.

Element	Role and proposition	Observable indicators	Would be disconfirmed by
Stage 1. Antecedent vulnerability	Probabilistic antecedent condition for uptake; selects exposure. Defined by criteria (a) to (d) in Section 6.2.	Dated pre-exposure clinical records, collateral reports, and communications showing psychosis, child-focused delusional content, conviction and affect, persistence.	Narrative-specific change and concordance at comparable rates in exposed offenders without Stage 1.
Moderator A. Pre-exposure narrative-state match	Moderates Stage 1 to Stage 2: match determines selection strength.	Blinded thematic match between documented pre-exposure content and published narrative content; no engagement evidence used.	Selection of focal narrative unrelated to match.
Stage 2. Selective exposure	Salient exposure, partly selected by Stage 1.	Dated search, viewing, and messaging traces; coverage volume.	Exposure in Stage 1 individuals no higher than in matched non-vulnerable individuals (selection claim).
Moderator B. Subjective identification	Moderates Stage 2 to Stage 3: increases encoding and rehearsal.	Dated statements of perceived similarity to actor or situation.	Objective resemblance predicts concordance equally with and without such statements.
Stage 3. Narrative uptake	Acquisition (new material) or activation (increased accessibility of existing representation); feeds back to Stage 2.	Acquisition: narrative-specific feature in post-exposure material, absent from adequately observed pre-exposure material. Activation: preregistered quantitative increase in rehearsal, searching, elaboration, immediacy, narrowing.	Concordance above base rate without any dated change (central proposition).
Stage 4. Appraisal and retention	Retrieved script retained or rejected by content congruence.	Dated post-exposure evaluative statements about the narrative in relation to own situation.	Dated change equally frequent at low and high pre-exposure match.
Stage 5. Behavioral selection	Retained script primarily shapes form; target fixed by Stage 1 is not evidence.	Base-rate-adjusted concordance on diagnostic features, scored against focal and control narratives.	Equal concordance with control narratives; concordance explained by vulnerability-fixed features.
Downstream narrative availability	Outside the model; cannot evidence pre-offense cognition.	Post-offense legal, clinical, and media framing.	Not a VSIM claim.

#### 6.2.1 Stage 1: antecedent vulnerability

Vulnerability is defined by clinical features that can be observed without reference to any later act, not by its effect on appraisal. In the developed model it is present when all of the following are documented in dated material: (a) a psychotic disorder; (b) delusional content that assigns the child a threatening identity, or that represents the child’s death as necessary, protective, or commanded; (c) high conviction, in the sense of Buchanan et al. (26), and affective response to the belief; and (d) persistence of (b) over more than one dated observation. Severity, treatment status, and functional impairment are recorded as covariates, not as criteria, because the literature does not support a threshold on them; they are expected to modify the probability of enactment (Stage 5) rather than of uptake. Three vulnerabilities that a single word conceals are kept separate: vulnerability to violence, which is the Stage A question of Section 7.1 and is not the construct here; vulnerability to narrative uptake, which Stage 1 as defined is hypothesized to confer; and vulnerability to enactment of a retained script, which depends additionally on proximal factors at Stage 5. Stage 1 is a probabilistic antecedent condition, not a necessary one; the term gating is avoided for that reason. Provenance rules apply: vulnerability counts only if documented in material dated before meaningful exposure and ascertainable without knowledge of the offense. A post-offense expert opinion does not qualify on its own. Pre-exposure violent ideation is not a vulnerability criterion, because it risks incorporating the outcome into the predictor; where present it shows that a relevant representation existed before exposure, and a dated post-exposure intensification of it may evidence activation at Stage 3. Acute intoxication is a proximal disinhibitor modifying Stage 5 only.

#### 6.2.2 Stage 2: selective exposure

The person is exposed to a publicized narrative under conditions of high salience: repetition, recency, vividness, emotional intensity, and active engagement. Stage 1 is expected to select exposure. Moderator A, pre-exposure narrative-state match, is a property of the pairing between the person’s documented state and the narrative’s content, coded before any outcome is scored, and determines the strength of selection. The arrow from Stage 1 to Stage 2 in **Figure 1** is unidirectional: the model does not propose that exposure changes antecedent vulnerability, only that vulnerability selects exposure.

#### 6.2.3 Stage 3: narrative uptake

The narrative either supplies new material (acquisition) or increases the accessibility of an existing representation (activation). Accessibility belongs to this stage. Moderator B, subjective identification and self-relevance, increases encoding and rehearsal here; objective resemblance raises its probability but is a proxy, and the suicide finding that effects are strongest where the observer resembles the person depicted (8) is read as evidence that resemblance facilitates identification. Rehearsal feeds back to Stage 2. Because forensic data rarely establish its temporal position, identification is a hypothesized process variable rather than a statistical mediator.

#### 6.2.4 Stage 4: appraisal and retention

The retrieved script is evaluated against the person’s current beliefs and goals and is retained or rejected. Content congruence at this stage determines retention. It differs from Moderator A in timing and function, and Section 7.4 gives the coding rule by which the two are distinguished empirically.

#### 6.2.5 Stage 5: behavioral selection

The retained script primarily shapes form. The distinction from motivation is one of primacy, not exclusivity: information about feasibility, consequences, or social meaning can alter urgency or inhibition. Where the antecedent vulnerability already specifies the victim, as delusions concerning the child do, the target is fixed by Stage 1 and is not evidence of scripting.

### 6.3 Central proposition

Because antecedent vulnerability can select exposure, a child-focused delusional system can independently produce both intense attention to filicide narratives and a child-focused act, and the two can look concordant without the exposure having altered the act. Resemblance alone is therefore weak evidence of influence. The strongest evidence exists when exposure temporally precedes a demonstrable change in narrative-specific behavioral information that is not parsimoniously explained by the documented antecedent state: under acquisition, a rare feature absent from adequately observed pre-exposure material emerges afterwards and appears in the offense; under activation, a demonstrably pre-existing representation shows a dated post-exposure increase in rehearsal, elaboration, planning, or behavioral priority without new content. Even this evidence is observational; the model claims strong support, not proof.

### 6.4 Prospective legal scripting and downstream narrative availability

After an offense, defendants, attorneys, clinicians, and journalists draw on available frameworks, and a recent trial that turned on postpartum psychosis makes those frameworks available for a later act. This institutional process concerns different actors, outcomes, and levels of analysis, and it would warrant a model of its own; it is placed outside VSIM (**Figure 1**) and used here only to mark what it cannot evidence. It cannot evidence pre-offense cognition. Prospective legal scripting, the incorporation of anticipated defenses into pre-offense decision-making, is an empirical possibility in principle, since psychosis does not imply global incapacity for instrumental reasoning and psychosis and legal insanity are different constructs; but it requires independent evidence of pre-offense anticipation, and a post-offense defense cannot supply it. The Yates testimony is the limiting case: an expert asserted that a media narrative had supplied both method and anticipated defense, and the narrative did not exist.

### 6.5 What VSIM adds and what it is

**Table 2** classifies each element as adapted or new. The adapted elements are the stage constructs and the moderators. The new elements are the three propositions stated in Section 1 and the evidentiary consequence that follows from them. The discriminating observation is this. A simpler model combining script theory, selective exposure, and delusion formation predicts that, among vulnerable people, exposure and concordance will co-occur. VSIM predicts the same co-occurrence but holds it uninformative and predicts in addition that influence will be detectable only as a dated post-exposure change in narrative-specific material; it further predicts that where such change is absent, concordance will be fully accounted for by vulnerability-fixed features and base rates. If concordance beyond base rates were routinely found among exposed vulnerable offenders without any dated post-exposure change, the simpler model would be sufficient and VSIM’s third proposition would be wrong. VSIM is therefore an organizing framework for existing constructs, an evidentiary protocol for evaluating alleged media influence, and a small set of propositions that make the protocol necessary.

**Table 2:** Provenance of VSIM elements: adapted constructs and new propositions.

Element	Status	Source or justification
Attention, retention, reproduction, motivation (background)	Adapted	16–18
Script accessibility, appraisal, rehearsal; acquisition versus activation	Adapted	19–22
Identification as moderator of imitation	Adapted	7, 8, 6
Selective exposure driven by existing state	Adapted	22 (rehearsal regulates affect); cognitive model of delusions (36)
Cultural shaping of delusional content	Adapted	31, 32, 34
Clinically defined, non-circular vulnerability construct with provenance rules	New (specification)	Section 6.2; criteria from 26
Proposition 1: vulnerability selects exposure, so exposure is not an independent variable	New	Section 6.3
Proposition 2: exposure primarily shapes form, not motivation	New (follows 4, made testable)	Section 6.2, Stage 5
Proposition 3: resemblance is uninformative; influence inferred only from dated post-exposure change	New	Section 6.3; discriminating observation in Section 6.5
Discriminant coding rules; estimand specification; separation of prospective scripting from downstream narrativization	New (protocol)	Sections 6.4, 7.1, 7.4

## 7 Estimands, predictions, measurement, and coding

### 7.1 Estimands

**Figure 2** gives the causal structure. Two estimands are distinguished. Stage A: does vulnerability predict whether severe violence occurs, and does exposure add to incidence? Stage B: conditional on an offense, does exposure change its form? VSIM is a Stage B model. Its target population is mothers meeting the Stage 1 definition, documented before exposure, who subsequently commit filicide; the comparison population is the same with low or no exposure to the focal narrative. The exposure contrast is high versus low engagement with one prespecified narrative within a window beginning at the narrative’s first prominent coverage and ending at the offense, with engagement measured from dated traces. The counterfactual outcome is the form the act would have taken had the person been unexposed, which is approximated by the form of matched offenses by unexposed vulnerable mothers and by the person’s own documented pre-exposure representation. The effect of interest is the interaction of exposure and vulnerability on form (a direct effect of exposure on form within the vulnerable stratum), not the total effect of exposure on offending. Confounders that must be measured are the latent state that may select exposure directly (addressed by the dated-change criterion rather than by adjustment), competing narratives (addressed by control narratives), and documentation availability, which affects observed exposure and observed vulnerability but not actual exposure or vulnerability; publicized cases are documented more fully than comparison cases, and this asymmetry must be modelled or matched. Cases without meaningful exposure enter the analysis as the comparison stratum and are essential; a design that includes only exposed offenders cannot estimate anything.

Conditioning on offense occurrence introduces collider bias, because vulnerability and, weakly, exposure both affect it (**Figure 2**). Offender-only designs therefore cannot estimate the Stage B effect without bias, and the long-term program requires vulnerable, exposed, non-offending comparison groups, which are obtainable only from clinical cohorts under care or from international registries. A single case meeting all evidentiary requirements provides strong case-level evidence of influence in that case; it does not validate the model.

**Figure 2:**
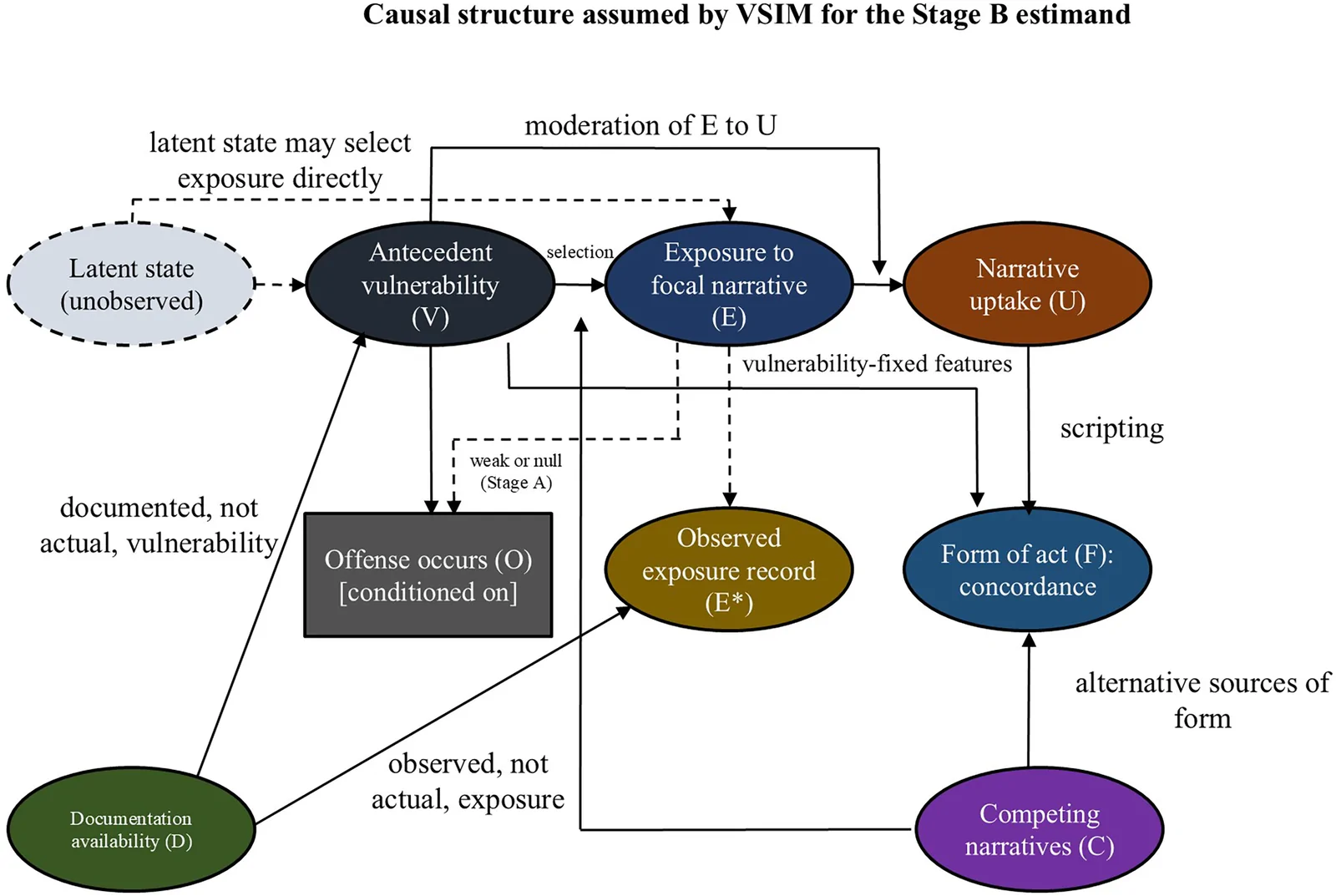
Causal structure assumed by VSIM for the Stage B estimand (form conditional on an offense). V, antecedent vulnerability; E, exposure to the focal narrative; U, narrative uptake; F, form of the act (concordance); O, offense occurrence; C, competing narratives; D, documentation availability; E*, observed exposure record. V selects E and moderates the E to U path; V also fixes some features of F directly, which are excluded from concordance scoring. A latent state may select E directly, which is why the dated-change criterion rather than adjustment is used. D affects observed, not actual, exposure and vulnerability, and is a measurement confounder. Conditioning on O (box), as offender-only designs do, opens a non-causal path between V and E and can bias the estimate; vulnerable, exposed, non-offending comparison groups are required.

### 7.2 Predictions

Each prediction states what evidence classifies its constructs, what evidence tests it, what would count against it, and what is inconclusive. Coding rules and analysis plans must be preregistered before any case is scored; this is a requirement, not a recommendation. The vulnerability and congruence constructs are defined by clinical features and thematic match coded without reference to outcome (Sections 6.2 and 7.4), so Predictions 1 and 4 are not restatements of definitions: they can fail.

#### 7.2.1 Prediction 1 (exposure by vulnerability interaction)

Classification: Stage 1 from dated pre-exposure clinical material; exposure from dated traces; form from the concordance profile. Test: the association between exposure and narrative-specific concordance is stronger among Stage 1 offenders than among matched filicide offenders without Stage 1. Against: an association of equal strength in both strata, or concordance among exposed non-vulnerable offenders at rates comparable to exposed vulnerable ones. Inconclusive: no exposed non-vulnerable stratum available. Corollary 1a (enrichment): offenders independently classified as exposed and showing dated post-exposure change will show Stage 1 more often than matched offenders without such change; this is supportive and not equivalent to the interaction.

#### 7.2.2 Prediction 2 (narrative-specific change)

Classification: acquisition and activation as defined in Section 6.3. Test: among exposed Stage 1 offenders, dated post-exposure change in narrative-specific material predicts concordance with the focal narrative but not with control narratives. Absence before exposure can be inferred only where pre-exposure observation was sufficiently intensive (regular clinical contact, continuous digital traces); otherwise, the acquisition branch is unavailable and only the activation branch, which requires a documented pre-existing representation, can be assessed. The activation indicators (search frequency, rehearsal in messages, method-specific elaboration, immediacy, narrowing of options) are fixed in advance, and no indicator not listed in the preregistration counts. Against: concordance with the focal narrative in the absence of any dated change, at rates above base-rate expectation; or equal concordance with control narratives. Inconclusive: no dated material in the exposure window.

#### 7.2.3 Prediction 3 (identification)

Test: among exposed Stage 1 offenders, dated statements of perceived similarity predict concordance beyond objective resemblance. Against: objective resemblance predicts concordance equally with and without such statements. Inconclusive: no communicative material.

#### 7.2.4 Prediction 4 (congruence)

Classification: Moderator A and Stage 4 congruence coded as in Section 7.4. Test: among exposed Stage 1 offenders, dated post-exposure change is concentrated where pre-exposure narrative-state match is high. Against: change at equal rates across match levels. This is not definitional because match is coded from narrative content and clinical content independently of whether change occurred.

#### 7.2.5 Prediction 5 (temporal profile; analogical)

The expectation that uptake tracks empirically measured peaks in exposure salience and decays as exposure and rehearsal diminish is imported from the suicide literature (8) and is not intrinsic to VSIM. Test: activation indicators and concordant offenses track measured exposure (coverage volume, social-media attention, the person’s own traces), with legal milestones only as examples of events that may generate peaks. Against: indicators unrelated to measured exposure intensity.

#### 7.2.6 Prediction 6 (population composition; secondary and analogical)

Because VSIM primarily changes form, incidence need not rise. The compositional outcome is the proportion of filicides, among those with a Stage 1 perpetrator, that are concordant with the focal narrative on preregistered diagnostic features, in a window of fixed length beginning at peak coverage, compared with the same proportion in matched unexposed windows. The test is an equivalence test against the smallest effect size of theoretical interest, prespecified and justified from the empirical distribution of concordance, measurement reliability, prevention relevance, and achievable precision, not extrapolated from the suicide literature. A pooled multi-national analysis capable of excluding that effect counts against the population-level prediction.

### 7.3 Measurement rules

Criterion independence: exposure and change are coded from dated traces and communications; concordance is coded from offense characteristics; different blinded coders handle the two. Antecedent narrative selection: the candidate is chosen from pre-offense traces by a preregistered hierarchy (explicit reference, then engagement intensity, then recency), never by thematic congruence with the vulnerability; where several candidates survive, each is scored, and control narratives matched on recency, prominence, and broad theme are scored for every case. Concordance: the metric is a future methodological requirement, not an operational component of the current model. Until it exists and is validated, “narrative-specific concordance” is a construct awaiting measurement, and Predictions 1, 2, and 6 cannot be run. **Appendix A** states what the metric must do, including sensitivity analysis to base-rate uncertainty, treatment of information missing not at random, and adjustment for the greater documentation of publicized cases. Offender-described rationale is excluded from the behavioral concordance score and analyzed separately as downstream evidence. Temporal ordering: vulnerability markers, exposure, change indicators, and offense are each dated.

### 7.4 Discriminant coding rules

Several constructs would otherwise be inferred from the same messages, searches, and records. The rules below assign each piece of evidence to one construct, and **Table 3** gives five hypothetical cases in which the constructs dissociate.

**Table 3:** Hypothetical cases in which VSIM constructs dissociate, with the coding each receives.

Case	Evidence	Coding
1. Match without identification	A mother with documented child-focused delusional content follows a filicide trial daily (dated traces) but her messages describe the defendant as “nothing like me” and “evil”.	Moderator A high (content match coded from records and narrative alone); Stage 2 present; Moderator B absent; Stage 4 rejection coded from the evaluative statements.
2. Identification without appraisal congruence	Messages state “she went through exactly what I am going through”; no statement evaluates the act, and later messages express horror at it.	Moderator B present; Stage 4 coded as rejection; no uptake coded.
3. Exposure without uptake	Extensive dated engagement with the trial; no new narrative-specific feature appears in any post-exposure material and no preregistered activation indicator changes; the later act shares only vulnerability-fixed and high-base-rate features with the narrative.	Stage 2 present; Stage 3 absent; Stage 5 concordance explained by base rates; no influence inferred.
4. Activation without acquisition	A documented pre-exposure representation of a specific method; after exposure, search frequency for that method rises fivefold and messages show rehearsal; no feature of the narrative absent beforehand appears.	Stage 3 activation coded on preregistered indicators; acquisition absent; influence supported on the activation branch.
5. Uptake without influence on selection	A rare narrative-specific feature appears in post-exposure messages (acquisition met); the later act does not contain it and matches the pre-exposure representation.	Stage 3 acquisition coded; Stage 5 concordance absent; prediction of form not supported for this case.

Moderator A (narrative-state match) is coded from two sources only: the clinical content documented before exposure and the narrative content as published. It is a property of a pairing and is scored without any reference to the person’s engagement with the narrative. Stage 4 (appraisal congruence) is coded only from dated material produced after exposure in which the person evaluates, endorses, rejects, or reinterprets the narrative in relation to her own situation. Match without such evaluative material is Moderator A only. Moderator B (identification) is coded only from dated post-exposure statements of perceived similarity to the actor or situation (“she is like me”); statements that evaluate the act (“she was right”) are Stage 4, and statements that merely attend to the case are Stage 2. Stage 2 (exposure) is coded from traces of attention and engagement with no required content. Stage 3 acquisition is coded only where a narrative-specific feature, absent from adequately observed pre-exposure material, appears in dated post-exposure material; Stage 3 activation is coded only where a documented pre-existing representation shows a quantitative increase on a preregistered indicator. A single document may supply evidence for more than one construct only if it contains distinct statements meeting distinct rules, and each statement is assigned once.

The research program is staged and low-risk: archival digital-trace studies; forensic interview studies of insanity acquittees extending Friedman et al. (30), with concordance coded blind to interview content; prospective observational studies of naturally occurring exposure in clinical cohorts under care, which also supply the non-offending comparison group; and analogue studies in non-clinical populations with non-violent scripts. International multicenter registries of psychosis-associated filicide, with linkage to clinical records and digital traces, would be the single most valuable infrastructure. **Table 4** gives candidate evidence and designs for each construct, and marks which designs measure constructs and which can identify the Stage B effect.

**Table 4:** Operationalization of VSIM constructs: candidate evidence, measurement, and whether the design can identify the Stage B effect.

Construct	Candidate evidence	Measurement approach	Design (M = measures construct only; I = can identify Stage B effect)
Antecedent vulnerability (Stage 1)	Pre-exposure clinical records; collateral reports; dated communications.	Criteria (a) to (d) coded from dated sources without knowledge of the offense.	Archival record review (M); registry linkage (M); clinical cohorts (M, and I when combined with unexposed and non-offending strata).
Narrative-state match (Moderator A)	Documented pre-exposure content; published narrative.	Blinded thematic match; no engagement evidence used.	Record review (M).
Exposure (Stage 2)	Search, viewing, messaging traces; coverage volume.	Frequency, recency, duration, engagement; antecedent narrative by preregistered hierarchy.	Digital-trace studies (M; I only with unexposed comparison); prospective cohorts (M, I).
Identification (Moderator B)	Dated statements of perceived similarity.	Coded separately from objective resemblance.	Forensic interview and trace studies (M).
Narrative uptake (Stage 3)	Dated appearance or intensification of narrative-specific material.	Acquisition and activation criteria, preregistered indicators.	Trace studies with intensive pre-exposure observation (M); cohorts (M, I).
Appraisal congruence (Stage 4)	Dated post-exposure evaluative statements.	Rule in Section 7.4.	Trace and interview studies (M).
Form and concordance (Stage 5)	Offense characteristics.	Metric meeting Appendix A; not yet available.	All case-level designs (M); pooled compositional analysis with unexposed windows (I, population level).
Downstream narrative availability	Filings, expert reports, coverage.	Discourse similarity beyond generic terminology.	Discourse analysis (M; separate model).

## 8 A worked example on a closed record: Yates v. State

The Yates case is used because it is fully adjudicated, because its forensic record is unusually complete and has been described by the defense expert (2), and because a claim of media scripting was made in it, tested, and found false (1). **Table 5** applies the model’s evidentiary rules to the record.

**Table 5:** Yates v. State as a worked example on a closed record: what the record shows against each VSIM element.

Element	Record (2, 1, 37)	Coding under VSIM
Stage 1. Vulnerability	Psychotic depression with hospitalizations and suicide attempts from 1999; delusional content concerning Satan and the children’s salvation; high conviction; persistence across clinical contacts over months before June 2001.	Met on criteria (a) to (d) from material dated before the offense.
Stage 2/Moderator A (prosecution narrative)	Expert testified to a Law and Order episode depicting a mother who drowned her children and was acquitted; stipulated that no such episode existed.	No exposure; no match. Claim of scripting unsupported at the first stage.
Stage 2/Moderator A (other media content)	Defense expert testified that Yates perceived personal messages from Satan in two feature films before the killings.	Exposure present; match present at the level of delusional theme, not of act.
Stage 3. Uptake	Content of films incorporated into delusional system; no feature of the act traceable to them.	Activation of delusional content arguable; no acquisition of narrative-specific behavioral material.
Stage 4. Appraisal	Films evaluated as being about her.	Congruence present.
Stage 5. Selection	Method, sequence, and target explained by documented delusional content and household circumstances.	No scripting inferred; form explained by Stage 1.
Downstream narrative availability	False media-scripting narrative introduced by an institutional actor, used in closing argument, accepted by the jury, and reversed on appeal.	Downstream process operating without any Stage 2 to 5 basis; illustrates the consequence of inference without the evidentiary rules.

Stage 1 is met on the published record. Yates had a documented history of psychotic depression with two suicide attempts and multiple hospitalizations beginning in 1999, well before the 2001 killings; delusional content that Satan was within her and that her children would be lost unless she acted; high conviction; and persistence across clinical contacts over months (2). Stage 2 and Moderator A are not met for the narrative the prosecution alleged: no such episode existed, so there was no exposure to be matched. For a different class of content, religious teaching and two feature films, the defense expert reported that Yates perceived personal messages directed at her, which is consistent with uptake of media content into a delusional system (37). On the model’s rules this is evidence of Stage 4 congruence (she evaluated the content as being about her) and arguably of Stage 3 activation, but it is not evidence of scripting, because the films did not depict the act and no narrative-specific feature of the act can be traced to them: the drownings, the sequence, and the target are explained by the documented delusional content and by the household. Stage 5 scripting is therefore unsupported.

The case illustrates three things. First, Stage 1 can be met rigorously from a real forensic record; the provenance rules are not utopian. Second, the record shows the incorporation of media content into delusional content without any media influence on form, which is exactly the dissociation the model requires an investigator to be able to recognize. Third, the Law and Order testimony is the downstream process in its purest form: an institutional actor supplied a narrative of media scripting, it was accepted by a jury, and it was false. Nothing in the model would have licensed the inference, because no dated exposure, no dated change, and no narrative existed. The example is not evidence for VSIM. It is a demonstration that the evidentiary rules can be applied and that their absence has had consequences.

## 9 Implications

### 9.1 Reporting: research questions, not guidance

Suicide is the only domain in which media imitation evidence has been translated into reporting guidance (42); analogous proposals exist for mass killings (43, 44). No guidance exists for filicide trials, and VSIM does not supply any. It generates three research questions. Does detailed reporting of method and sequence in filicide trials increase activation indicators in exposed Stage 1 individuals under care? Does reporting that emphasizes treatment and recovery reduce them, as the Papageno literature would predict (7)? And is any such effect detectable at all given the base rate? Until these are answered, no reporting recommendation follows from the model. One clinical distinction must accompany any public message about help-seeking: unwanted, ego-dystonic intrusive thoughts of infant-related harm are common, occur in perinatal anxiety and obsessive-compulsive disorder, and are not associated with aggression toward the infant (45–47). They are categorically different from psychotic delusions concerning the child.

### 9.2 Clinical practice: constraints

One statement is independent of VSIM and rests on established practice: the emergence of delusional content assigning a child a threatening identity, or representing the child’s death as necessary or protective, warrants urgent assessment, because child-focused delusions are clinically concerning features documented in psychotic pathways to filicide (15, 30, 40). The model adds no recommendation to this. It does not establish preoccupation with a publicized case as a risk marker, and the following constraints apply to any future use. The false-positive rate of media preoccupation as an indicator would be very high, because engagement with notorious cases is common and filicide is rare; an indicator with that profile cannot be incorporated into actuarial or structured risk assessment without validation, and none exists. Questioning about media engagement in a patient with child-focused delusions risks suggestibility and confirmation bias, since the clinician’s hypothesis is transparent and the patient’s reasoning is impaired; it also risks therapeutic trust, since the patient may correctly perceive that ordinary interest is being treated as evidence of dangerousness. And any clinical uptake of the model risks reinforcing the inaccurate public association between psychosis and violence. Media exposure is hypothesized as an organizer of form, not as a risk factor, and the model’s clinical content is, for now, nil.

### 9.3 Possible extensions

Reviewers of theory in this area reasonably ask whether the stages transfer to mass shootings and grievance-fueled homicide, which attract intense coverage and in which offenders demonstrably study prior offenders (43). The stage structure and the evidentiary rules are expected to transfer. The vulnerability construct is not: grievance is not a clinical state, has no equivalent of the conviction and persistence criteria, and would require its own non-circular definition, probably in terms of dated expressions of a specific injustice and a specific target population. The appraisal mechanism would also differ, since grievance narratives supply legitimation rather than threat. These are extensions to be built, not claims of the developed model.

## 10 Limitations

VSIM has not been tested. Its target phenomenon is rare, and the full model cannot be tested without international registries or archival digital-trace designs that supply both exposed and unexposed Stage 1 offenders and non-offending comparison groups. The vulnerability construct is developed for one pathway, and its criteria (b) through (d) rest on the delusions-and-violence literature rather than on filicide-specific evidence. The concordance metric does not exist, and until it does the model’s central outcome is a specification rather than a measurement. The dated-change criterion mitigates but does not remove confounding by a latent state that could generate both a rare idea and attention to a narrative containing it, and it is available only where pre-exposure observation was intensive, which will exclude most cases. Publicized cases are documented more fully than comparison cases, and documentation availability is a measurement confounder that must be addressed by matching or modelling. The evidence base is largely analogical, and Section 2.6 states what it can bear. VSIM is a model of how form is transmitted; it is not an etiological account of filicide, of psychosis, or of violence.

## 11 Conclusion

Severe violence does not ordinarily create models that others follow. What a publicized act can do, in a person whose antecedent state has already made such violence conceivable, is supply form. Because that state also selects what the person attends to, resemblance between two acts is weak evidence of influence; the inference requires a dated change in narrative-specific material that the state does not explain. The Yates record shows both that the antecedent state can be rigorously documented and that a claim of media scripting, asserted without that standard, can be false and consequential. VSIM supplies the standard.

## Data Availability

The original contributions presented in the study are included in the article/supplementary material. Further inquiries can be directed to the corresponding author.

## Appendix A. requirements for a concordance metric

This appendix states requirements; it is not an instrument, and the model cannot be tested until an instrument meeting these requirements has been built and validated on offenses with known exposure histories. (1) Profile dimensions are fixed in advance: method and method-specific features, victim age, setting, temporal pattern relative to measured exposure, sequence, symbolic or scene-specific elements, and pre-offense communicative features; victim relationship, features fixed by documented vulnerability, and post-offense rationale are excluded. (2) Reference distributions are estimated from national filicide data stratified by perpetrator sex and victim age, with uncertainty in base rates carried through to the score and reported; rare-feature base rates will be imprecise, and apparent rarity may reflect incomplete reporting. (3) Coding is blind to exposure evidence, with preregistered rules, inter-rater reliability, and preregistered handling of missing values; because missingness is unlikely to be at random (publicized cases and cases with digital traces are documented more fully), sensitivity analyses under plausible missing-not-at-random assumptions are required, and documentation volume is recorded as a covariate. (4) Rare matched features receive greater weight than common ones through a function established and validated empirically; candidates include information-based weights, likelihood-ratio approaches, and comparison with empirically generated reference distributions. Naive additive inverse-base-rate weighting is not assumed: it is unstable for rare features and double-counts correlated dimensions, and dependence among dimensions must be modelled. (5) Scores for the prespecified narrative are compared with scores for control narratives and with scores obtained by matching the target offense to random reference filicides. (6) Features meeting the acquisition or activation criterion are flagged separately.
